# Production and reception of human resource management practices for health promotion

**DOI:** 10.3389/fpsyg.2023.1104512

**Published:** 2023-03-30

**Authors:** Beatriz Cintra Storti, Marina Greghi Sticca, Amalia Raquel Pérez-Nebra

**Affiliations:** ^1^Laboratory of Organizational and Work Psychology, Department of Psychology, Universidade de São Paulo, Ribeirão Preto, Brazil; ^2^Social Capital and Wellbeing Research Group (BYCS), Department of Psychology and Sociology, Universidad de Zaragoza, Zaragoza, Aragón, Spain

**Keywords:** human resource management practices, managers, teams, mental health, social support, universities, well-being

## Abstract

Organizations thrive when there is a healthy relationship between people, i.e., where there is high social capital. Human resource management practices (HRMP) contribute to promoting social capital and mental health in organizations. However, there remains a gap in the literature on practices to promote mental health, as well as on the difference in perception of the function of the practices between those who promote them and those who receive them. Thus, this study aimed to identify what HRMP oriented toward mental health promotions are, how they are perceived, and whether there is variation among these perceptions. Twenty managers and 11 subordinates were interviewed. To achieve the first two objectives, a content analysis was performed, and for the last, a lexical analysis. In the content analysis, the following categories emerged for both groups: work organization and idiosyncratic deals and affective social support. Only in the managers did the categories of informational support, communication, and maintaining good interpersonal relationships emerge. The lexical analysis suggested that managers perceive task-related practices as promoting mental health, while teams attribute importance to affective social support practices. HRMP psychological principles were described. Social support practices should be adopted as human resource protective strategies for mental health.

## Introduction

High levels of social capital in organizations have been associated with better mental health and well-being rates among individuals (Clausen et al., 2019). Moreover, promoting health and well-being for all (United Nation Organization, 2015) is among the United Nations Sustainable Development Goals (SDGs). The 2030 Agenda sets out to lead the world on a path of resilience by promoting sustainable development under the guidance of five principles: people, planet, prosperity, peace, and partnerships aiming for the global well-being of this and future generations (Sianes et al., 2022). Organizations (Serafini et al., 2022) and particularly universities for their educational, scientifical, technological, and innovative character (Bal et al., 2019) are among the actors responsible for achieving these goals. Among the goals of SDG 3, which aims to “ensure healthy lives and promote well-being for all at all ages” is the promotion of mental health and well-being (United Nation Organization, 2015). Considering that the SDGs require the efforts of organizations of various kinds and of universities to achieve them (Sianes et al., 2022), this goal can be better accomplished in work environments where people trust each other and help each other, i.e., where there is social capital. The prevalence of social capital in organizations makes them more likely to thrive, and it contributes positively to increased well-being and reduced rates of absence (Kiss et al., 2014) and suicide among their employees (Diener and Seligman, 2004). Human resource management practices (HRMP) gather some evidence as one of the ways to foster social capital in organizations and increase workers’ well-being and mental health (He et al., 2019); however, it lacks typification (Bowen and Ostroff, 2004) and understanding of its function, even more so in non-WEIRD (Western, Educated, Industrialized, Rich, Democratic; Henrich et al., 2010) cultures (Wang et al., 2020), presenting cultural distance (Muthukrishna et al., 2020), and less supportive contexts (Pérez-Nebra et al., 2021a,b) as Brazil.

The work in universities is shrouded by aspects that have led to the compromising of the health and well-being of its workers, with increasing rates of Burnout Syndrome and mental problems (Levecque et al., 2017), also with evidence in Brazil (Cassandre, 2011; Carlotto and Câmara, 2017). According to Bal et al. (2019) efforts to improve the work environment should begin within the university itself, since these institutions have been established around an unhealthy system that has been making people sick all over the world. The authors suggest the need for human resources protection practices, given the importance of such actions in crisis contexts (Bal et al., 2019). Thus, this study identified which HRMP promotes mental health, how these practices are perceived, and how they vary across actors. To test its proposition, the article presents the Brazilian university context, a literature review that explores the characteristics of work in universities, the impacts that these aspects have caused on workers’ mental health, and recent studies on HRMPs in view of their potential for promoting mental health in organizations.

### The university context in Brazil

Work in universities is immersed in a system that drives overload, competitiveness, and scarcity, and puts the mental health and well-being of academics at risk globally due to the burnout that results from exposure to these factors (Bal et al., 2019). Their activities are diverse and permeate teaching and research as well as administration. In addition to producing knowledge, they also engage in funding opportunities and other managerial services that lead them to play multiple roles: they are writers, teachers, collaborators, administrators, researchers, conveners, supervisors, leaders, entrepreneurs, negotiators, and advisors (Subban et al., 2022).

Among all these demands and their effects on the mental health of academics is the logic of the “publish or perish” system, which evaluates academics through their publications, and the overvaluation of metrics that measure the performance of these professionals, which leads them to search for limited spaces to publish their work and incurs competition. In addition, the current publishing model resembles a “business” in which journal editors use the free labor of academics, leading to professional burnout (Bal et al., 2019). Currently, countries such as the United States, China, and Germany, in this order, are at the top of the ranking that quantifies the world scientific production (CGEE, 2021) and as a consequence of this logic that involves the work of academics, studies conducted around the world, including these countries, show that university workers are feeling themselves increasingly pressured and show signs of Burnout Syndrome, anxiety, and psychological stress, in addition to demonstrating job insecurity (García et al., 2016; Carlotto and Câmara, 2017; Tian and Lu, 2017; Cladellas-Pros et al., 2018; Bal et al., 2019).

In Brazil, as in many other countries, the number of publications in international scientific production has grown considerably in recent decades. The scientific production had a 32% increase between 2015 and 2020 that placed it in the 13th position of world production of scientific articles indexed in the Web of Science base. This performance singled out Brazil in isolation from the other Latin American countries (CGEE, 2021), as Brazilians publish many more articles than the other countries in this region (Web of Science Group, 2019). Yet, the resources invested by the Brazilian government in science and technology have been known worldwide as an issue for years, as they have, conversely, decreased (Meis et al., 2003; Web of Science Group, 2019). These data are important, because besides being part of the non-WEIRD countries, where studies on people management practices are rare (Bonache et al., 2012), the expressive academic production that the country presents at a global level (CGEE, 2021) and the scarcity of resources directed to Brazilian public universities (Meis et al., 2003) that reflects in the working conditions made available (Souza et al., 2020) and in the mental health of Brazilian academics (Cassandre, 2011; Carlotto and Câmara, 2017; Kanan et al., 2018), add up as factors of relevance for the exploration of the theme through this study. This contraposition intensifies the margins for competitiveness in universities, due to the scarcity of resources and the precarious conditions that make it difficult for academics to work (Souza et al., 2020) and make them must work increasingly hard to survive professionally.

Because they are inserted in this competitive and demanding environment, the daily work of Brazilian academic professionals is surrounded by several factors that can harm their mental health, leading to stress and psychological distress (Kanan et al., 2018). In addition to the excessive pressure for publications that makes the reported system of “publish or perish” operate in a biased manner (Diele-Viegas et al., 2022) and surprisingly perceived as useful and fair (De Queiroz et al., 2020), the highly bureaucratic processes, the need to work with scarce resources, the expectations for results, the constant updating of technological changes, as well as the diversity of the students’ profile, the search for constant improvement in the quality of education, the demand for internationalization, the absence of support, as well as the increase in external control that has led to a discussion about the autonomy of universities, are also configured as risks that compromise the mental health of these professionals (Cassandre, 2011; Wilhelm and Zanelli, 2013).

Although performance and the metrics used to evaluate work are important for the success of organizations, their shortsighted prioritization in universities at the expense of other types of performance and the well-being of academics should be considered (Bal et al., 2019). The health harms caused by the current management system at universities make the performance of workers unviable and unsustainable. In contrast to the obsessive paradigm of performance, the literature points to High Well-being Model as a model that can contribute positively to workers’ health, as it fosters a culture of collective happiness and improves people’s well-being through aspects such as flexibility, optimism, trust, commitment, security, and learning (Ravina-Ripoll et al., 2019), which are vectors that also promote social capital. The HRMP can play a prominent role to develop the High Well-being Model, through the mechanisms that constitute it and mitigate the impacts caused by universities on the mental health of workers, as they contribute to positive organizational changes amidst the crisis taking place in academia (Bal et al., 2019). However, despite its relevance for Brazil due to the worrying scenario about the mental health of universities workers, the studies found in the literature on HRMP reveal that there is a concentration in samples from North American, European, and Asian countries, while in Latin America its frequency is scarce, performance-oriented (Oliveira et al., 2023) and, above all, theoretical. This imbalance limits the possibilities of guidance on the effectiveness of HRMP in countries located in this region (Bonache et al., 2012) and can also be problematic due to the impact that cultural, social, economic, and value differences between countries can have on managerial behavior and, consequently, on the HRMP adopted in organizations (Lenartowicz and Johnson, 2003).

### Human resource management practices

The origin of the studies in the HRMP area are predominantly from WEIRD countries located in North America, Europe, and Australia, it is still the case that often the results of these research studies are generalized to a broad population while in fact they refer to only around 12% of the world’s population (Henrich et al., 2010). This makes the conclusions that researchers reach about work-related human behavior not necessarily useful and representative to non-WEIRD countries (Muthukrishna et al., 2020; Pérez-Nebra et al., 2021a,b), such as Latin American (e.g., Brazil), because these nations have their unique historical development, which may lead them to resist the imposition of foreign HRMPs, such as those of the Anglo-European model (Posthuma et al., 2014). Thus, the prevalence of the lack of research on HRMP with this population specifically, makes the knowledge and theoretical and practical contributions regarding the topic insufficient for most of the world, since they do not fit into this small slice of research.

Over the past three decades, models of HRMP applied in organizations have explored the association between practices and employee performance, commitment, and engagement (Bal et al., 2019). However, the purpose of HRMP has been changing, and with it their priorities in terms of goals. The possibility of mutual gain between the organization and the employee is sought, and while mutual gain is not an outcome, it may be a priority goal (Guest, 2017). Furthermore, despite the knowledge achieved about the contributions of HRMP to the promotion of well-being (He et al., 2019), it is still unclear what these practices are, how they are perceived by managers and employees, the possible divergence of function, and how this occurs in a work context where the physical and mental health of employees has been put to the test, as in the case of Brazil (Pérez-Nebra et al., 2021a,b).

When using the term HRMP, the literature refers to the smallest part of the human resource management strategies and policies implemented by organizations and defines them as the routine actions, the daily activities that allow such policies and strategies to be executed (Martín-Alcázar et al., 2005). The literature review conducted by Van Beurden et al. (2020) on employee perceptions of HRMP suggests that they are related to three perspectives, depending on their focus, and reported more broadly, these being: social exchange, communication, and occupational health. These perspectives are supported by theories that ground the allocation of HRMP based on different conceptual models.

In exchange relationship practices, organizations provide incentives to people and people contribute to organizations based on how they perceive those incentives and reciprocate (Van Beurden et al., 2020). Social Exchange theory claims that interpersonal relationships are basically composed of costs and rewards, i.e., people offer each other material or immaterial goods and services that entail a cost and expect to receive a reward in return. These are the actions of the individual, who is motivated to do them by the expectation of the other’s reward for his action, which is usually fulfilled. Reciprocity is the main premise of the theory, since it presupposes the acts of giving and receiving (Blau, 1964). Social Exchange Theory can be verified in the indicators of people’s engagement at work, which is directly linked to the HRMP adopted by organizations. Thus, when people perceive that they are valued, trusted, and treated as partners, they respond positively to organizations through their work engagement (Tensay and Singh, 2020; Teo et al., 2020). Linked to the social exchange theory, the Equity Theory (Adams, 1965) studies the factors that lead to the perception of fairness or unfairness in exchange relationships and how people feel affected when they perceive unfair outcomes. As such, the theory assesses the fairness of these outcomes, which is done by comparing the individual’s costs with those of another person in similar situations and the rewards obtained by each. Therefore, they negatively impact their behaviors which could be favorable to organizational outcomes if people perceive the practices as fair (Madison et al., 2018). The Equity Theory also contributes to the understanding of psychological contracts, since they resemble each other in terms of the expectation for exchanges due to the consideration given by an individual to another party (Rousseau, 1989). Psychological contracts originate from individual beliefs in a reciprocal obligation between the individual and the organization and arise from subjective perceptions and unwritten expectations between the two. They are tied to the individual’s commitment to the organization and act as an important determinant of people’s work behavior (Rousseau, 1989). To HRMP are important for the development and cultivation of psychological contracts, and so is investigating people’s perceptions of these practices, because when they are perceived and evaluated positively, due to the sense of belonging and appreciation they trigger, they contribute to work engagement, since they lead to people’s need to reciprocate what the organization offers them (Katou and Budhwar, 2012; Soares and Mosquera, 2019). Finally, the person-environment fit theory describes the match between people (their needs, values, and goals) and the characteristics of the organizational environment in which they are embedded (rewards, job demands, and cultural values) (Kristof-Brown et al., 2005). Alignment between the parties benefits both organizations and employees and positively influences their attitudes and behaviors. Furthermore, research suggests that HRMP are conducive to making this adjustment happen, and empirical results have already been found in line with this statement (Boon et al., 2011; Mostafa and Gould-Williams, 2014).

Communication practices are related to what and how organizations communicate with their employees (Van Beurden et al., 2020) and are usually oriented to clarify what the social norms are and what is expected of each party, providing coherence and consistency (Mortensen and Cialdini, 2010; Miller and Prentice, 2016). Bowen and Ostroff (2004), in the strength of the human resource (HR) management system model, described the characteristics of a HR management system that cooperate for a strong organizational climate, in which people share a common interpretation of expected and rewarded behaviors, which when reproduced, collaborate to organizational performance. The model is based on attribution theories (Kelley, 1967) that help identify the characteristics that lead to communication messages between employees and the organization to be interpreted uniformly from causal explanations to behaviors and situations according to their degree of distinctness, consistency, and consensus. The higher these degrees, the stronger the HR management system (Bowen and Ostroff, 2004), because “it enhances clarity of interpretation which allows similar cognitive maps to develop among employees and creates an influence situation that causes employees to understand and yield to messages regarding appropriate behavior” (Gill et al., 2018, p. 312). In relation to HRMP, attribution theories contributed to the creation of the concept of HR attributions (Nishii et al., 2008), which refers to the reasons organizations adopt HR practices. Research points out that employees perceive and interpret in different ways the same HRMP deployed by organizations (Hewett et al., 2018), and that these attributions relate distinctly to aspects such as performance, commitment, and satisfaction depending on how the practices are attributed (Shantz et al., 2016). The social information processing theory (Salancik and Pfeffer, 1978) may help understand the difference between these interpretations since it explains the process under which the understanding of communication and the environment occurs. In this model, the social component exerts a strong influence on individuals’ attitudes, which become products of the processing of the information to which they have access (Zalesny and Ford, 1990). Therefore, people’s perceptions of their surroundings vary, because they result from how they interpret such information and thus make sense of what happens around them (Jiang and Li, 2018). Finally, the signaling theory also helps to understand individuals’ perceptions of HRMP, since it seeks ways to reduce the information asymmetry between the senders and receivers of information through signaling (Spence, 2002). That is, in organizations, the theory can be transposed by considering that practices are the signals that managers (senders) send to employees (receivers), and the way these signals are sent (frequency, channel, modality) can influence the perception of employees and, consequently, the organization’s communication (Wang et al., 2020).

Occupational health practices are interpreted from the demands and resources that exist from work, the more resources there are (material, social, personal, etc.), the more likely the negative effects of work on people’s well-being will be reduced (Van Beurden et al., 2020), and the basic principle guiding these practices is that of resource scarcity. Three models are related in this category: (1) Job Demand-Resources (JD-R) by Demerouti et al. (2001): this model classifies job characteristics as demands or job resources, with demands requiring effort and being associated with possible compromises in workers’ health, while resources contribute to work engagement and to an intrinsic and extrinsic motivational process (Demerouti and Bakker, 2011; Bakker and Demerouti, 2017). Considering this premise of the JD-R model about work arrangements as a mechanism for providing resources, HRMPs are also configured as such, because through their implementation workers’ needs are more likely to be met and the effects of this application are perceived through employees’ engagement and performance with work (Stirpe et al., 2022); (2) Job Demand-Control (JD-C) by Karasek (1979), in which control is defined according to the extent to which the individual has decision making about his or her work in relation to skill use (i.e., learning, repetitiveness, creativity, task variety, skill development) and the autonomy for their own decision-making and; (3) Resource Conservation Theory (RCT), which posits that people are motivated to protect their current resources and acquire new resources, and these vary across individuals and are directly linked to their personal experiences and situations (Halbesleben et al., 2014).

Thus, the study starts from the hypothesis (H1) that HRMP’s perspectives of social exchanges, communication, and occupational health (Van Beurden et al., 2020) will be found in participants’ descriptions.

According to Guest (2017), models of HRMP are still oriented toward increasing performance and workers’ issues are still a secondary issue in the models. Therefore, the prioritization of workers’ well-being and health is put into focus only when perceived to affect performance. It turns out that different reviews already present poor results of the relationship between well-being and performance (García-Buades et al., 2020; Peiró et al., 2021; Pérez-Nebra et al., 2021a,b) negatively impacting the prioritization of practices focused on the care of workers’ health and well-being. However, considering that well-being is a result of human, social, and ethical interests, besides being a bridge to performance, it is necessary to seek work that offers dignity to the human being (Bal, 2020), pursuing practices oriented to health, care, and quality of life, among others, including within universities, where precarious working conditions and their harmful consequences on the mental health of workers around the world have already been identified (Carlotto and Câmara, 2017; Levecque et al., 2017; Tian and Lu, 2017; Cladellas-Pros et al., 2018).

Boon et al. (2019) reviewed several scales of measures on HRMP and found that most were performance-oriented practices and that scales oriented to incorporate protective practices or foster mental health or social capital in organizations are rare. The authors distinguished practices in their levels of analysis between intended, implemented, and perceived practices. The practices reported by managers usually show what is intended and what is actually offered in organizations; however, when employees make this report, they report how they perceive and experience these practices, which opens up room for differences to be observed between these actors’ perceptions and discourses about HRMP (Boon et al., 2019). The difference between practices implemented and perceived by managers and employees is already noted in the literature, including due to the attributions made by employees regarding their role (Beijer, 2014; Woodrow and Guest, 2014) which, because they are divergent, may cast doubt on the effectiveness of their implementation and their associated outcomes.

Thus, it is hypothesized (H2) that managers’ perceptions of the role of HRMPs identified differ from those of teams (Boon et al., 2019; Van Beurden et al., 2020) and (H3) the differences between these perceptions are statistically significant.

Given that HRMP contribute to the promotion of people’s well-being (He et al., 2019; Bal, 2020) and that the results found on HRMP in non-WEIRD countries also make up this demand, actions related to working conditions, which are linked to the well-being and health of workers, require greater investment. This need can be extended to higher education institutions, since research on the topic in this environment represent only 4% of all sectors analyzed, which may suggest a lack of knowledge about practices aimed at promoting the health of these workers (Demo et al., 2018).

In this sense, once the contribution of HRMP to the well-being of workers is verified, with a focus on the promotion of mental health and social capital in public universities, a greater proximity to the theme becomes necessary. Thus, in this study, it will be possible to identify which HRMP have been perceived in these organizations to contribute to the mental health of workers. This study also proposes to test three hypotheses: (1) the perspectives of social exchanges, communication, and occupational health of Van Beurden et al. (2020) study will be found across types of HRMP; (2) managers’ perceptions of the role of HRMP identified differ from the perceptions of the teams and; (3) the differences between these perceptions are statistically significant. To test these hypotheses, which are still mostly limited in non-WEIRD countries, exploratory qualitative research proves to be the appropriate approach for this investigation.

## Methodology

### Study design and sample

The approach used for the research was qualitative, which means that the research aimed to investigate the perceptions and experiences of the participants on the investigated topic.

Semi-structured interviews were conducted between the months of May to August 2022 to access detailed and in-depth information on HRMP adopted and perceived in the context of the participants’ work that contributes to promoting employee mental health. The study sample was composed of 20 educational managers who worked within Brazilian public universities, 12 men (60%) and 8 women (40%), in different positions, 16 (80%) at the tactical level (i.e., course coordinator) and 4 (20%) at the strategic level (i.e., pro-rector) and 11 members of these managers’ teams (i.e., professors and technical-administrative secretaries). The interviewees were from different regions of the country, 3 from the state of São Paulo (33%), 4 from the state of Minas Gerais (44%), 1 from the state of Ceará (11%), and 1 from the state of Goiás (11%).

To fit the sample criteria, the managers had to have been working in management positions on the date of the interview for at least 3 months and had to have at least one employee under their responsibility. Regarding team members, they should also have worked in the manager’s team for at least 3 months. Initially, the participants were invited to participate in the study by means of individually forwarded e-mails accessed through the websites of several public universities in the country. Due to the low return on these e-mails and the difficulty of access to university managers, those who responded and agreed to participate in the research were asked at the end of the interview to indicate the contact of other fellow managers of the same or other universities so that the invitation to participate in the research could be made to them. Also, after each interview, the managers were asked to indicate the contact details of at least one member of their team so that data could also be collected by this part of the sample.

We used an interview script based on a model of HRMP and mental health promotion validated by two judges, represented by the first two authors of the article, whose choice criteria were based on their contributions to this production. Thus, after the script was structured and validated by the first judge, the second judge also evaluated it to avoid possible biases in the established questions, limitations, or ambiguities that could affect the answers’ quality and the participants’ understanding. In case of disagreements, a third judge, represented by the third author of the article, would be invited to evaluate the interview script, but this process was not necessary at this stage. Therefore, each interview involved researchers and participants engaging in an informal, casual conversation, exploring each participant’s personal experience of HRMP and mental health promotion.

Participants received information about the study and gave written consent before the interviews were conducted. Data on age, sex, time in the organization, and job title were also collected on the consent form to report the demographic details of the sample. All interviews were recorded, online, and completely transcribed.

### Measures

#### Interview script

Two semi-structured interview scripts were developed based on the dimensions proposed by Van Beurden et al. (2020), relating the questions to management and mental health practices, one for managers and another for teams. At this stage, content validation was performed with 2 expert judges in the field who received the digital document and provided feedback on the semantic validation, quality, and purpose of the questions. The judges requested the inclusion of a definition of well-being to improve the understanding of the questions and proposed changes in the format of some questions to avoid laconic answers. We also conducted a pilot study with 5 managers to check for understanding and comprehension of the terminologies and the clarity of the questions. These managers were recruited by convenience (through ease of access by the researcher) but had to meet the following criteria: they had been working in the public sector in a management position for at least 3 months and had at least one subordinate linked to their management. It was found that the participants in the pilot interviews had difficulties relating the characteristics of the work and the practices they adopted in management to their effects on the mental health of the team, so some changes were made in the questions to facilitate this understanding. For example, changes were made to broader questions (e.g., it was explained what the term “mental health” referred to, citing the interference of work in aspects such as mood, emotions, thoughts, and reactions of the participants concerning situations exemplified by them that impacted their mental health, and examples of the situations experienced were requested to describe the human resource management practices adopted to promote employee mental health, asking about daily actions taken by them that made the team feel less anxious, more relaxed).

The focal questions included in the managers’ interview script explored (1) task characteristics and impacts on mental health (e.g., What are the main activities developed by your team? Do you perceive that the activities performed by your team impact their mental health?); (2) management practices and mental health (e.g., Which human resource management practices do you adopt to minimize work impacts and promote the team’s mental health?).

The focal questions included in the team members’ interview script explored (1) task characteristics and impacts on mental health (e.g., What are the main activities developed by your team?; Do you notice if any of the activities you perform at work impacts your mental health?); (2) management practices adopted by managers and mental health (e. g., Which human resource management practices does your manager adopt to minimize work impacts and promote your team’s mental health?).

### Data collection

Initially the participants were invited to participate in the study by individually forwarded e-mails that were accessed using the websites of several public universities in the country.

The interviews were conducted online *via* Google Meet, and the interview script was previously sent *via* email to each participant before the interviews, to facilitate their approach and preparation. We also sent a formal invitation *via* e-mail. With the participants’ consent, all interviews were audio-recorded. Each recording was transcribed with the help of a piece of software to simplify the process and verify the total reliability of the information collected.

In the case of the staff interviews, from the beginning the participants were again informed about the secrecy and confidentiality of their answers. This was done to minimize the risk that their reports may be biased by the fact that it was their manager who referred them to participate in this study and, therefore, they could be subject to possible harm as a result of their answers if they were unfavorable to the performance of the manager as to the HRMP adopted.

### Data analysis

#### Categorical content analysis

All interviews were manually transcribed in their entirety and categorical content analysis according to Bardin’s (1977) hypotheses was used to test H1. This analysis was divided into three stages: First, the data was reread to acquire more familiarity with the content collected and then to identify all the HRMP mentioned by the participants. This information was separated by tabs for the organization of which practices were mentioned by the managers and which were mentioned by the teams. This first organization led to the creation of an initial category called “general practices.” The frequency with which each HRMP appeared in the narratives of the managers and teams was quantified.

In the second stage, the practices that emerged from the interviews were grouped together and the categories created from this process were named in line with the management competences to which they were related. For example, the practice identified and described as “being available to talk, listen and intervene on the team’s demands” was grouped with the “affective social support” competence.

Finally, the last grouping that originated the final categories of the HRMP identified in this content analysis had the categories proposed by Van Beurden et al. (2020) as theoretical support, including social exchange, communication, and occupational health, fundamentally oriented toward the function of the practice for the belonging group. Therefore, social support for one group may be based on reciprocity and for another, on occupational health, depending on the repercussion and the individual’s expectation discourse about the behavior.

The content analysis of the managers’ narratives led to the identification of six intermediate categories of HRMP adopted by the managers to promote the team’s mental health. Five of these categories were grouped into three final categories, which were: idiosyncratic deal (social exchange); communication, and affective social support (communication), and work organization and informational support (occupational health) and friendliness. The categories of HRMP identified by the teams’ narratives were: work organization (occupational health); idiosyncratic deal (social exchange), and affective social support (communication).

For this classification, the study also counted on the participation of the two mentioned judges, who had simultaneous access to the data collected and cross-checked them to compare the categorizations performed with each other and avoid biases, detect omissions and ensure constancy (Gibbs, 2009). Disagreements that emerged between the two judges were solved by convoking a third judge (and third author of the article) who also evaluated the organization of the categories. After collective discussions, decisions about the final categorization were made by consensus among the judges.

#### Lexical analysis

We transcribed all the interviews literally. After the transcription, we organized the corpus. We did it by standardizing the Portuguese language and connecting keywords. For example, mental health had to be rewritten as mental health.

The lexical analysis was conducted using Iramuteq and the Camargo and Justo (2018) Iramuteq protocol. We conducted 31 interviews, with 366 segments, 12,583 occurrences, and a total of 676 hapaxes. We also conducted Reinert Classification with Descendent Hierarchical Classification (DHC) and Correspondence Factor Analysis (CFA).

#### Lexical analysis comparison

To compare the classes of the lexical analysis with both clusters, we conducted a chi-square analysis, which allowed us to identify the differences between groups. It was a similarity analysis conducted on an absence/presence of the group, which crosses the selected units in a row and the active forms of the class in a column. Those differences were considered significant when the test is greater than 3.84, based on 1 degree of freedom and *p* < 0.05. This analysis was used to test hypotheses 2 and 3.

### Results

The categorical content analysis led to the organization of the results under the categories of HRMP from the study by Van Beurden et al. (2020). This result corroborates H1, suggesting that the study’s three dimensions would be found in the HRMP cited by the participants. In the managers’ narratives, the practices of affective social support and communication were allocated in the “Communication” category. The informational support and work organization practices were related to the “Occupational health” category, and the idiosyncratic deal practices were framed in the “Social exchanges” category (Table 1). The practices identified by the teams’ discourse were: work organization (related to the “occupational health” category), affective social support (related to “communication”), and idiosyncratic deal (related to the “social exchanges” category) (Table 2).

**Table 1:** Human resource management practices adopted according to the managers’ perception.

Human resource management practices adopted according to the managers’ perception					
Final categories	Intermediate categories	Initial categories	*n*	*f*	Examples of narratives
Communication	Affective social support practices	(1) Be available to talk, listen and intervene on the team’s needs*	1	7	“Being on their side, showing trust, partnership, relying on them, on the capacity they have, and (…) definitely counting on us.”
		(2) Promote situations on a daily basis to socialize with the team*	2	3	“There are the moments when we involve the whole team to set up a Christmas tree, organize a festivity. In find that has a very positive impact.”
	Communication practices	Maintain clear and transparent communication, with cordiality and politeness**	3	4	“Then I called the teacher, listened to everything he had to say, and I was very calm like, ‘try to do a little bit differently here’.”
Social exchange	Idiosyncratic deal practices	Substitute classroom teachers*	4	1	“If the teacher is going to be late, I go to the classroom. He tells me the subject he is going to deal with and I go and talk to the students until the teacher arrives.”
		Give the team autonomy at work	5	4	“They have total autonomy, I like to stress, as long as there is no conflict with the university regiment or statute.”
		Provide flexibility in working hours and schedules*	6	2	“If he can't go in a given time it’s okay, because that’s it, he's not a person who doesn't perform.”
		Assisting/instructing them to perform specific tasks*	7	2	“They ask: can’t you extend the deadline? And we say: no, we need it, it must be fast. We have already done this before, I will send you the spreadsheet that I’ve done, and you fill it in.”
		Share the demands to solve them with the team to create a sense of belonging*	8	1	“I always ask, ‘what would you do?’ And people feel more like they belong to the management and not just as the role doers, but as the ones who will solve the problems.”
		Be available to talk, listen and intervene on the team’s needs*	9	1	“You give space, you respect. Of course there are moments of exchange, there are moments that I have to listen and there are moments when they have to listen to me.”
Occupational health	Work organization practices	Arrange for interns/staff to assist in task distribution	10	2	“We have interns to help. We try to get more staff to help them.”
		Guide them to delegate what is possible / discard unimportant tasks		1	“Whatever activities we can discard we discard, and I tell them to outsource whatever is not pertinent to them.”
		Promote improvements in health resources and physical workspace		1	“We are implementing an agreement with a university here in town that has a psychology course, to deliver preventive group courses.”
		Centralize decisions for you / Filter the demands to the team / Reduce the number of meetings	11	3	“I don’t overburden them. Collegiate meeting I call only once a month, when I do. Only when I have to decide something quite big, otherwise, if I can decide alone, I decide.”
		Do not send messages “after hours” and on weekends (and tell them not to answer)	12	3	“WhatsApp can be bursting with messages when you’re off, you don’t have to answer. No one will be bothered on the weekend.”
		Saving psychologically vulnerable people from the most stressful activities	13	1	“She has already said she can’t, so we’ll go. I have already agreed with the academic director to send it to me, to spare her, not to send it to her.”
		To be flexible on deadlines, deliveries and schedules	14	3	“I ask: 'You will be responsible for doing this. Then the person says, 'When?' I say, ‘Yesterday, but I understand that’s not possible. How much time do you need?’”
		Replace teachers in the classroom*	15	1	“In the case of this teacher who forgot her class, she called me, apologized, and I said, ‘it's okay, it happens, go rest. I was there, so I took over her class.”
		Making working hours and schedules more flexible*	16	1	“There was one person who had a miscarriage and it was very difficult. She had a leave of absence, but it wasn’t enough for her to get well. So I didn't give her the same deadlines as the others, but that wasn’t disclosed to the team.”
		Share the demands to solve them with the team to create a sense of belonging*	17	1	“We always try to talk to him to try to make him understand that it’s not all his responsibility.”
	Informational support practices	Improve team work processes and instruments to lighten related demands	18	1	“We improved the forms to fix some issues so that there wouldn't be so much demand for something that could be solved with the provision of a rule.”
		Assisting/instructing them to perform specific tasks*	19	2	“I try to share the task, including myself, being part of the whole.”
		Provide the necessary information for the team to solve their demand	20	2	“I tell him where he can look for, that there will be a person who will have that information for him.”
		Help them prioritize tasks / set limits on demands / make a delivery schedule	21	3	“That’s what I’m trying to do. She really understands that it’s not like having 5 or 6 demands coming in and they all have to be answered simultaneously. Prioritize and go.”
		Respond to staff as soon as possible, even outside working hours or through personal contact	22	1	“I don’t get upset about having to respond after hours, as I know it’s an urgent demand, I try to resolve it for them as quickly as possible.”
		Maintain clear and transparent communication, with cordiality and politeness*	23	1	“I try to be a friendly person with her. I try to spare her from certain issues.”
Friendliness	Maintaining good interpersonal relations	Be available to talk, listen and intervene on the team’s needs*	24	1	“In two years, I won’t be there anymore (…) I will be a teacher who will demand from him as the others do (...) He has a sofa there, God forbid, I’m almost calling the cleaning crew to clean it. Other coordinators have already told him off about this. He said: what’s up, professor? I said: what I need is for you to do your job. What you do during your break is no problem. Every administration has its own style, and this is mine.”
		Promote situations in daily life to socialize with the team*	25	1	“I have tried a socialization in non-work actions. There is a very close relationship of assigning tasks, this is part of management. And to try... I consider myself a relatively sociable person and I’m also talking about very pleasant people, it’s no effort for me when I bring my coffee here for us to drink together, because I think this closeness strengthens.”
		Replace teachers in the classroom	26	1	“In every individual meeting I have with a professor, I try to show that I have empathy for him or her. I arrive to talk to him as a co-worker, exposing the situation (...) I can exchange time with a teacher, give up my class, replace a professor.”
		Centralize decisions for you / Filter the demands to the team / Decrease the number of meetings	27	1	“They all ask me to be the manager again. So I feel that they like me, they like the way I do it (...) I have professors that participate in other collegiates, and they tell me “I can't stand collegiate meetings anymore”. And I don’t do this kind of thing anymore. So, I think they realize that I hang in there a lot, so as not to get to them. And I think this contributes in a way.”
		Assisting/instructing them to perform specific tasks*	28	1	“I often say that we have to try to do for people what we would like them to do for us (...). I try to share the task, including myself, being part of the whole.”

*Practices perceived by managers with different functions and therefore allocated in more than one category. *n* refers to the number of practices found and *f* the frequency of description of those practices.

**Table 2:** Human resource management practices adopted according to the teams’ perception.

Human resource management practices adopted according to the teams’ perception					
Final categories	Intermediate categories	Initial categories	*n*	*f*	Examples of narratives
Occupational health	Work organization practices	Requests more personnel to assist the team	1	1	“She seeks, they made this request for more technicians, so now the Dean’s office knows that we are in a big deficit.”
		Promotes improvements/ achievements to the physical work space	2	1	“I stayed in a precarious room, but with a lot of struggle my room finally came out and it was my manager who helped provide it.”
		Assists the team in demands/doesn't delegate everything*	3	2	“Many things he does too, he doesn’t leave everything to us, so I think that helps a lot too.”
		Intermediates/ Delimits the demands and situations of the team’s work*	4	1	“He buys our fights, like: ‘such and such must be done and it is urgent'. He says: ‘no, we are not going to do it, you can put it under my responsibility because there is no way.”
		Respects/ provides flexible schedules and deadlines*	5	1	“He worries about this issue of respecting schedules. Because it is a position of trust, we have to be available to meet demands if they are urgent. But he respects this issue.”
		Shows empathetic, open to dialogue/ team support*	6	1	“The coordinator went through some health issues. She got sick to the point she didn’t want to hear about the college. So there was a change in the administration, and the deputy chief couldn't be reappointed because he was in another position, but he took over the responsibility from the coordinator, and even though he is deputy chief, he is the one who solves everything.”
Communication	Affective social support practices	Shows empathetic, open to dialogue / team support*	7	4	“She is very supportive, any problem you can talk to her, she is empathetic, she says: we'll find a way, don't worry. She doesn't make you feel guilty.”
		Intermediates/ Delimits the demands and situations of the team’s work*	8	1	“When a student’s problem comes up and I can't solve it, besides her having a different outlook for being outside the problem, when she helps me solve it, I feel her support.”
		Respects/ flexibilizes schedules and deliveries*	9	3	“But, who is going to apply this activity? Sometimes we can’t, and then the coordination is available. Of course everyone is required to have their own schedule, to perform their own activities, but we have flexibility and that is very good.”
Social exchanges	Idiosyncratic deal practices	Gives feedback/ recognizes the work done	10	2	“We get together sometimes, he gives me some very gentle nudges. But how does he help me? We talk about my reports, and I take some of his suggestions.”
		Gives autonomy/ demonstrates trust in the team	11	3	“She said: You don't even have to tell me, if you think it’s a task that you can pass on to them, do it. You don't even have to go through me.”
		Communicates clearly and objectively so as not to prolong meetings	12	1	“Whenever we have meetings, we already have a pre-established agenda, it is very succinct, objective, and this makes our lives easier with such a long workload.”
		Assists the team in demands/doesn’t delegate everything*	13	2	“Several times during that period, she did my turns. I have to deliver the diploma of the graduates of the program, and since I have comorbidity, she never let me do that.”
		Intermediates/ Delimits the demands and situations of the team’s work*	14	1	“Whenever we talk to her, she says: ‘we'll find a way, I'll talk to the specialized care center that works with psychological health.”
		Shows empathetic, open to dialogue/ team support*	15	5	“She calls beforehand, tries to find out individually if we can, and we go to the meeting with things already more or less defined. And we also have time to think, sometimes we even change our minds, decide to take on what we didn’t want before.”

*Practices perceived by the teams in different ways and therefore allocated in more than one category. *n* refers to the number of practices found and *f* the frequency of description of those practices.

The results show that a total of 28 managers’ narratives practices, the occupational health category, which is related to reducing demands and/or increasing work resources, emerged with greater variability of practices (*n* = 14) and a reasonably distributed frequency among them. Practices related to work organization were the most cited (*f* = 17) in the category. Among the communication practices in this group, those linked to affective social support, and particularly the practice of “being available to talk, listen and intervene about the needs of the team,” obtained the highest frequency among the managers (*f* = 7). Finally, in the category of social exchanges, the practices related to trust and the autonomy that the manager claims to give to his team were seen by these actors as favorable to the mental health of their employees (*f* = 4).

In relation to the team practices’ narratives (n = 15), it was the category of social exchange that emerged with the greatest variability of practices (n = 7) and also with the highest frequency of responses (*f* = 17). Social exchange practices refer to the negotiations and agreements that are made between managers and employees based on the costs and “efforts” of both parties expecting rewards for these actions that are usually voluntary. Their prominence in the teams’ narrative may suggest a representativeness of this group’s perception regarding how they see these practices contributing to the promotion of their mental health.

Another interesting result of the content analysis was that, in addition to the functions of HRMP provided in the categories according to Van Beurden et al. (2020), something also emerged from the managers’ narrative that seems to be intended to make the employees “like” them due to the practices adopted to contribute to the promotion of the team’s mental health. Although in relation to their frequency the function of these practices is incipient, this finding led to the creation of the category Friendliness for the allocation of these practices (which were framed in more than one category), since Van Beurden et al.’s (2020) typification between practices of social exchanges, communication, and occupational health, would not support this other function found for some of the same practices already previously classified.

The Descending Hierarchical Classification (DHC) organized the lexicons into three classes. The presentation of the results of this analysis also allowed the naming of the classes in line with the dimensions of HRMP of Van Beurden et al. (2020). Class 1 was named “Task support” because the words that emerged are related to practices whose focus was on the tasks of the team’s work. Class 2, “Social exchanges,” was so named because it highlights words that suggest practices regarding negotiations between managers and teams. Class 3, “Affective social support,” was so named because the emerging lexicons relate to HRMP focused on the interpersonal aspect between the two groups. The inference made between the actors’ narratives and the types of practices most frequently cited by each of them also allows us to relate the results of the content analysis to those of the descending hierarchical classification.

**Figure 1:**
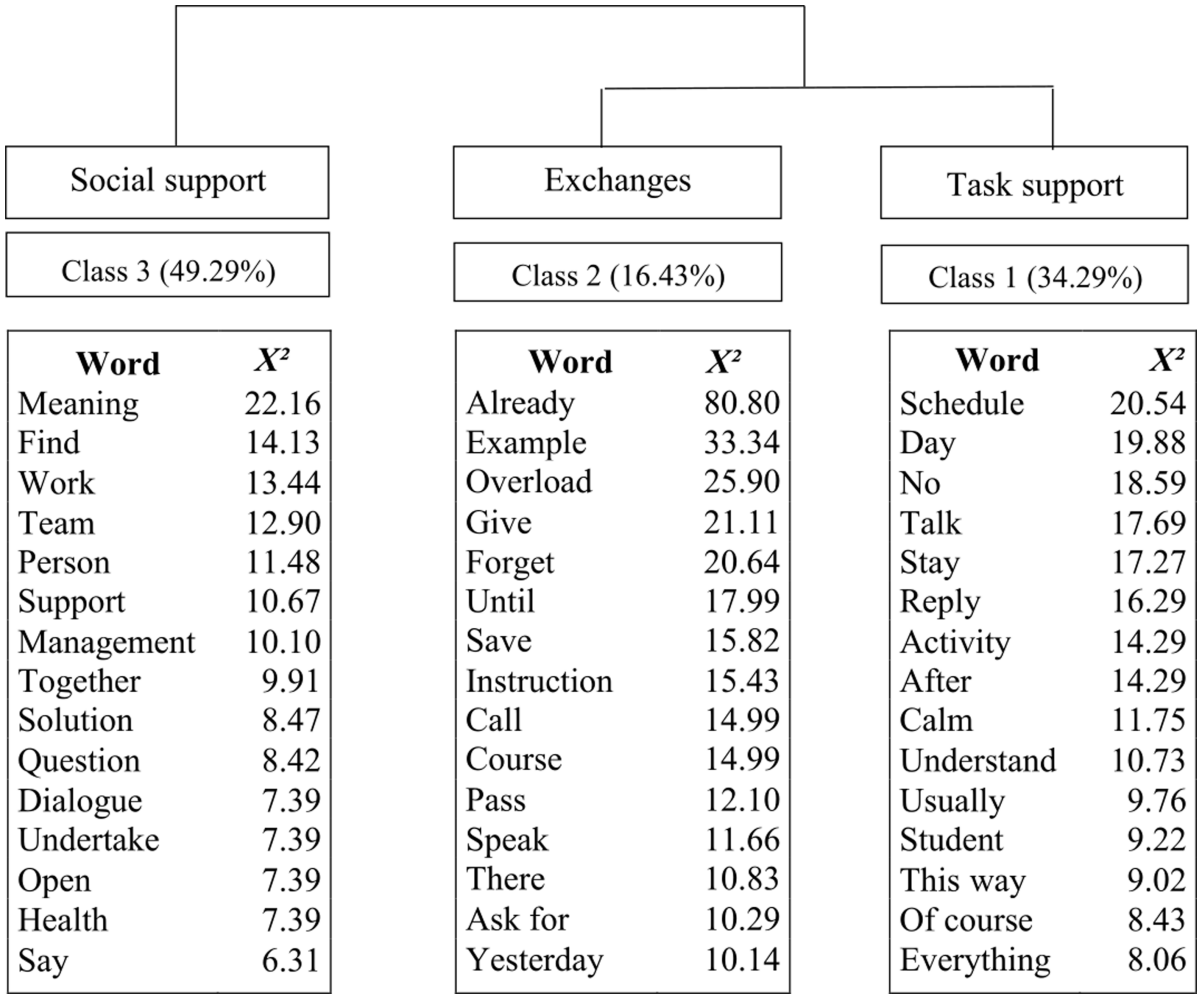
Descending hierarchical classification (DHC) dendogram.

The words emerged in the lexical analysis show proximity between Class 1 and the category Occupational health of the content analysis. This means that in both analyzes, the participants’ narratives were more directly related to the practices adopted by managers through the acquisition of resources and/or the reduction of demands of the teams’ work aiming at the promotion of mental health of these people. The same occurs with Class 2 and the category Social exchanges, as well as with Class 3 and the category Communication. In Class 2, the lexicons suggest an exchange relationship between the interviewed groups, and the flexibility practices that fall into this category demonstrate the negotiations and agreements established between managers and teams that are seen positively by them in terms of mental health. The adoption of these practices leads to a perception of reciprocity and, consequently, expectations and a kind of Commitment to be followed by both parties through these exchanges. Finally, the Class 3 results are linked to Communication practices since they suggest in the discourse of the groups the notion of social norms, care and support, which favors the social environment by providing support and a sense of unity, and thus increases the chances of positively influencing the attitudes and behaviors of people within organizations because of how this support can be perceived in terms of protecting and promoting mental health (Figure 1).

There was a difference between the results identified in the content analysis and the descending hierarchical classification regarding the category of Social exchange. Although in the DHC the results of this category were not significant, in the content analysis its frequency was expressive, especially in the narratives of the teams. Moreover, as suggested in H2, a difference was found in the actors’ perception regarding the typification of the practices mentioned. Table 3 shows some examples of narratives from each category. These narratives are extracted from Iramuteq, which provides verbal reports in descending order of the most representative excerpts of each class of words.

**Table 3:** Narratives related to each class.

Classes	Examples of narratives
(1) Task support	“He respects this issue very much, so he understands very well that given our schedule here, we also need to engage in other activities, and not get too attached to the work here.”
	“We try to take in what is requested and also try not to bring more problems. We also try to understand them, talk to them, try to give them an understanding of what is happening in general, because this suffering is not only theirs.”
(2) Social exchanges	“The coordinator is a manager who avoids handing over too much to others. I think she even overloads herself a lot, I’ve already told her too, but she spares us a lot.”
	“We had two professors who had their doctoral defenses. Theoretically, the institution does not oblige me to give them a day, but I say: ‘people, for God’s sake, we have already been through this. So we gave her the previous day off.”
(3) Affective social support	“Within my management, what I have done is when we identify a teacher who is ill as a result of work, we take a collective measure in the sense of sparing the person.”
	“Valuing people. I try to do this because I think it is important. I think that if the person is here to do a job, he or she is competent. Sometimes what they need is support, someone to be by their side.”

**Figure 2:**
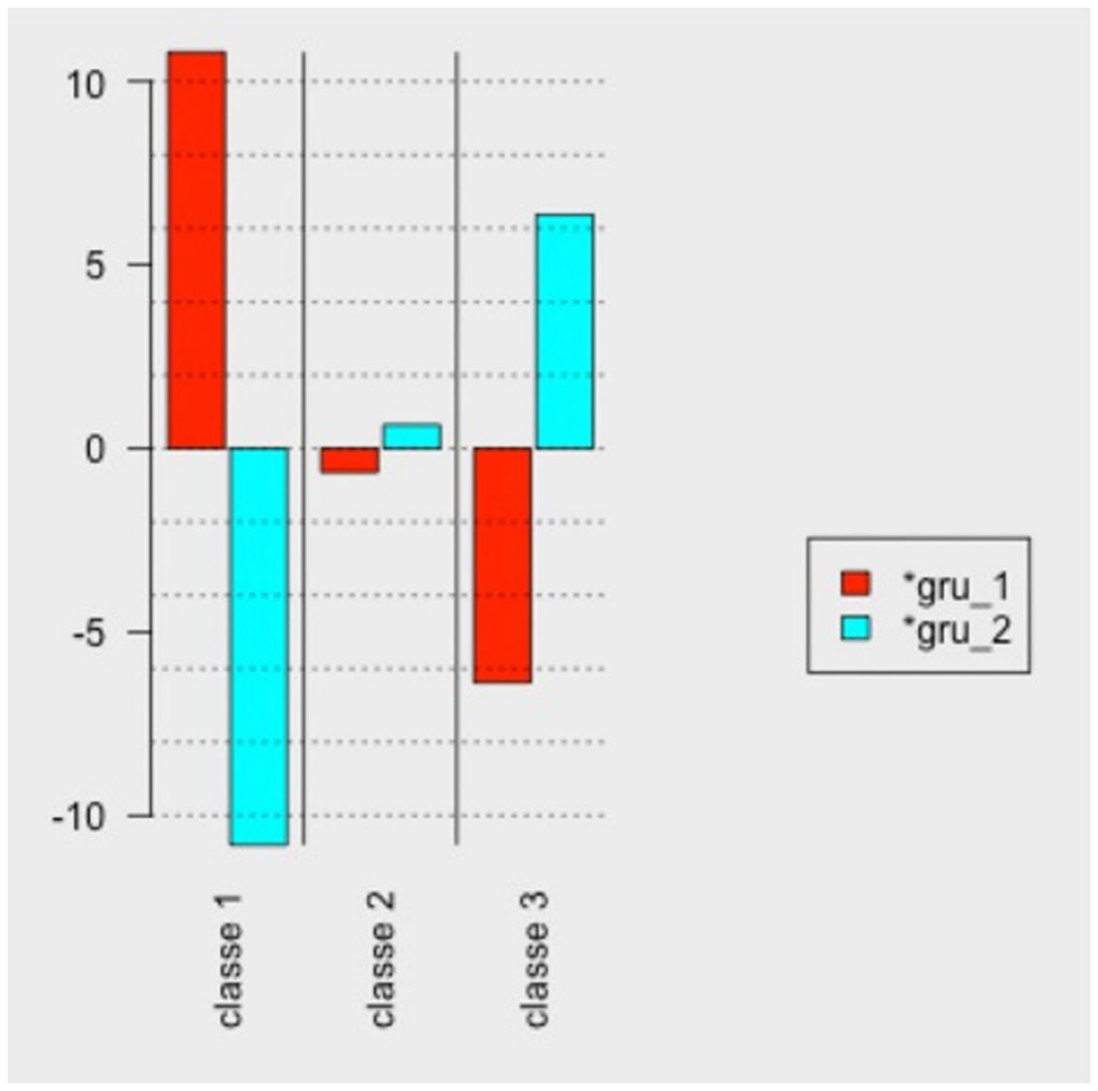
Chi-square of the difference between groups and lexicon classes.

The difference analysis between groups suggests that there were significant differences between Classes 1 and 3, with Group 1 (managers) showing higher frequency in Class 1 - Task support, and Group 2 (teams) showing higher frequency in Class 3 - Affective social support. Class 2 - Social exchanges - did not show significant differences (Figure 2). Thus, the results of this analysis give partial support to H3.

## Discussion

This study aimed to identify HRMP that contribute to the promotion of mental health of workers in Brazilian public universities and it is understood that this objective was met. Three of the four categories of practices that emerged in the study were typified according to the study by Van Beurden et al. (2020), that is, as exchange relationship practices, communication practices, and/or occupational health practices supporting H1. Furthermore, there was variation between managers’ and employees’ perceptions of these practices again supporting H2. In addition to the fact that some of them were mentioned in the narratives of one group and not in the narratives of the other, the function that these practices fulfilled also presented variations among the participants in relation to the same practice. Finally, not all classes of the descending hierarchical classification presented significant differences, offering partial support to H3.

Previous studies pointed out that specific management practices, defined through managers’ concrete behaviors, contribute to the management of work stressors on managers’ and staff’s mental health because they predict well-being and overall health (Bouchard and Meunier, 2022). Three principles emerge as relevant to thinking about HRMP: the principle of reciprocity, consistency, and scarcity. These psychological principles have been widely examined in the field of social psychology (e.g., Petty and Briñol, 2012) and marketing (Cialdini, 2009) and emerged among the categories presented by Van Beurden et al. (2020). It is noteworthy, however, that in the case of managers in Brazil, there is another principle that emerges transversely, which is linked to the principle of liking, i.e., the manager strives to make people like him or her and thereby wants to do something for this person for the simple fact to be liked and prefer positions which “they think would be ingratiating and avoid those that would make them look bad” (Petty and Briñol, 2012, p. 234). This principle has been reported before in the literature of HRMP, usually coming from the worker, a tactic called ingratiation (Ferris et al., 1999; Bolino et al., 2008), but also friendliness as a component of success for the manager in some cultures (Smith et al., 2012).

From this perspective, the category Friendliness that emerged from the managers’ narratives received empirical support. In the case of this study, the friendliness principle, especially, was found in the context in which managers report the search for a partnership with the collegiate, since “everyone is in the same boat” concerning the challenges experienced within public universities and that “today they are managers, but tomorrow they will be teammates again,” due to the turnover of professors who have to take over management positions. Cialdini’s (2009) friendliness principle says that individuals, in general, prefer to say yes to the demands of people they like. In this sense, by adopting practices that are “approved” by the teams, managers are more likely to receive acceptance and friendliness from their collegiate members. From this standpoint, the practices classified in the categories of social exchanges and communication (especially in relation to social support), seem to be rather close to the category of friendliness, since the function and intentionality with which each practice is applied can even be confused with the manager’s need to “be liked” - although this does not cancel out the other possible contributions of the practices cited. As an example, it is possible that this dynamic is related to the statements found in the managers’ narratives about a certain “avoidance” of resisting, hindering, or not making certain concessions to professors both in relation to work and to personal demands - as shown in the intermediate category of idiosyncratic deal - having in mind, on the other hand, the possible idea of the manager that “if tomorrow this teacher is my manager, I could also be harmed.”

Regarding the results of the teams, which highlighted the role of social exchange practices through what we categorized in this study as Idiosyncratic deals, we verified the perceived importance of negotiating adaptations and customizations in their work according to their needs, which, therefore, was seen as favorable to the promotion of mental health. Although they are not *per se* a HRMP, idiosyncratic deals seem to “blend in” and be favored by them, besides benefiting individuals and organizations. On the one hand, since it is a proactive behavior, which usually starts from the employee to the supervisors, the results derived from the HRMP adopted (i.e., autonomy, motivation, engagement, commitment) enable this attitude of employees that resonates in terms of performance for the organization. On the other hand, the pursuit of these negotiations exposes the active role of employees in HRMP in relation to seeking to adjust job characteristics to meet their needs (Villajos et al., 2019; Latorre et al., 2021). Therefore, the findings of this study seem to offer some support that the application of idiosyncratic deals and their relationship with exchange practices lead to a win-win situation between managers and teams. However, the literature related to idiosyncratic deals and well-being (in which mental health is included) is still incipient, and what we see is that the main variables studied in association with this construct are still predominantly related to performance, as already found by Boon et al. (2019) in terms of practices.

Finally, the comparison between groups showed statistically significant differences between the groups in Classes 1 (task support) and 3 (affective social support). The variation in the actors’ perceptions of HRMP and their role reported in this study, corroborates with the literature that already pointed to this possible divergence (Beijer, 2014; Woodrow and Guest, 2014; St-Hilaire et al., 2018; Boon et al., 2019). In the managers’ view, it is the practices related to occupational health that contribute to the promotion of employee mental health. On the other hand, the teams consider that the practices of exchange and communication (in particular, social support) bring this contribution the most. For managers, the practices that emerged among the most cited seem to be related to what we categorized in this study as occupational health (i.e., task support, workload management, working time, and holidays). For subordinates these practices also stood out, but those suggesting affective social support were more frequent (i.e., valuing and recognizing work, interacting, initiating relationships, dialoguing and promoting participation, disclosing, empowering, representing). The findings converge with the results of the Descending Hierarchical Classification that found the relevance of reducing demands and/or increasing resources in the view of managers, and the contribution of the sense of team (communication practices) on the part of employees for the promotion of mental health.

The convergence between the findings of the present study and the results of St-Hilaire et al. (2018) are interesting and lead to another possible reading, in that the considerable frequency with which practices related to social support appear in both, while for managers the reduction of demands is mostly seen as able to promote mental health. It is possible that the place from which the managers “speak” to the teams, closer to information about the limited resources available in public universities, makes them seek in the reduction of these demands a way to mitigate the impacts of the work on people’s mental health, wherever possible. However, the teams bring in their report the awareness that the manager usually has few tools to offer more resources and balance these demands, and consequently, they recognize a more effective help in situations in which he is willing to give an even affective support to the team.

There is evidence that the implementation of HRMPs can strengthen employees’ well-being (Sora et al., 2021), health, motivation and skills, bringing benefits to both individuals and organizations, as they result in higher productivity and less sick leave (Ybema et al., 2020). However, the results pointed out above reinforce the need highlighted in previous studies (Van Beurden et al., 2020), about the importance of listening to employees in search of understanding about the functions that these practices fulfill for them, because it is their perceptions about the practices that influence their behavior and, therefore, will obtain the expected potential in promoting their mental health. Consequently, if these perceptions are not aligned, as the actors’ narratives suggest, the likelihood of the practices not fulfilling their possible contributions will be increased and their effectiveness, on the contrary, reduced. Thus, in addition to not effectively contributing to the promotion of the mental health of the team, the adoption of HRMP misaligned to the needs of individuals may even have an effect contrary to the goals of organizations because the more aligned to the needs of individuals, the more likely the HRMP will positively influence the engagement and proactive behavior, but when this adjustment does not occur, engagement decreases and exhaustion levels increase (Van Beurden et al., 2022), which also influences employee well-being.

The presentation of the HRMP identified in this study also leads to the suggestion of what are the possible psychological mechanisms that link these practices to their results, i.e., that make them protective to the mental health of employees. What can be inferred from our findings is that, beyond the differences found between managers and teams, there is a consensus on the perception by managers that such practices may, and, really seem to produce the feeling of belonging, welcoming, trust, recognition, consideration, partnership, exchange, and unity in the perception of individuals. Therefore, these are the aspects that become “the fuel of the practices,” the mechanisms by which they are remembered and mentioned by the individuals as favorable to the promotion of their mental health. Thus, the importance given by managers and teams to show their peers that they are together, that the well-being of one is important to the other, and that they can support each other, reflects the characteristics of collectivist cultures such as Brazil (Hofstede, 1984; Gouveia and Clemente, 2000; Smith et al., 2012). In them, individuals are led to think collectively, seek harmony, value communication, relationships precede the priority of tasks, management is done for groups, and the relationships between employee and employer are seen even as a family bond (Hofstede, 1991), as suggested in several excerpts of the interviewees’ narratives.

The circumstances of the context of Brazilian public universities presented by the literature for years have already pointed out the risks that this environment offers to the mental health of workers, mainly due to the high demands on the work and performance of academics in the socioeconomic scenario where these organizations are located, which go against the provision of various resources that could assist in achieving these expectations in a healthier way (Cassandre, 2011; Wilhelm and Zanelli, 2013; Kanan et al., 2018). Although they were not the focus of this investigation, living with these risks permeates the discourse of the interviewees, who are invariably reminded when talking about the impacts of work on their mental health. In the midst of this problematic, HRMP such as those cited in this study emerge as a simple, but effective possibility to mitigate the impacts caused by the working conditions of Brazilian public universities on people’s mental health. Although they should not be considered as the only solution for all the shortcomings that universities face, the essentially collaborative aspect that guides most of the HRMP adopted is potentially protective to the competitive and often solitary environment in which academic work takes place, which can be mitigated, with some evidence, by the care with people within by the management.

### Practical implications

This study contributes to the sustainable performance of academic managers by presenting which HRMP adopted in their daily work contributes to mental health promotion in universities. Therefore, access to the practices identified and detailed through concrete behaviors as done in this study, can assist managers in efforts to promote staff mental health from their daily actions (Bouchard and Meunier, 2022).

In addition, studies that have conducted this type of investigation have found that the practices adopted focus more on performance than on people’s well-being and mental health (Boon et al., 2019). In this sense, from the intersection of the perceptions of managers and teams, this study also makes practical contributions to this existing gap in the literature on HRMPs aimed at promoting the mental health of university employees, because, in addition to identifying them, it also specifies the functions that such practices fulfill when they are implemented. This enables them to be more effective in relation to outputs, as it avoids both misalignments between how they are intended and received, and the waste of resources spent by universities for them to be adopted - which is also relevant, given the problematic shortage of resources for years present in Brazilian public universities (Meis et al., 2003; Souza et al., 2020).

### Limitations and future directions

From a practical standpoint, strategies can be developed to allow managers to develop social exchanges more effectively. Within the workplace, the literature points out that individual strategies such as emotional regulation (Kim et al., 2014) can help improve manager and team health. Moreover, although previous studies have been devoted to investigating employees’ perceptions of HRMPs implemented in organizations, the typification, principles, and functions they fulfill to people are still unclear.

Social support practices, widely cited in the health literature as protective strategies for physical and mental health, emerge again as important strategies with different functions in the organizational context. It is worth mentioning that although they occur, the systematization of these practices is still incipient and depends on each dyad and the idiosyncratic deals. In addition, although ingratiation has emerged as a HRMP, it requires care not to be confused with self-promotion. The literature on impression management suggests that joking can be beneficial, but caution is needed, particularly in a collectivist culture where if perceived as false, “liking” can be harmful and have a reverse effect to that expected.

The most frequent practices are those oriented toward reciprocity and the longer and more mutually contributing to the relationship, the higher perception that a growing social capital is built in the organization. Higher education institutions in the Brazilian context comprise a group that has been living together for several years and the scarcity of public contests in universities and of resources has generated a context of competitive strategies, segregation patterns and an absence of management and inclusion of the difference (there are no policies related to affirmative actions beyond those provided by law). Detecting HRMP that are protective to the group will foster social capital and a more solid public institution in the medium long term, consistent with the goal of sustainable development 3 and 16 to promote good health and well-being and peace, justice and strong institutions.

Finally, whether they have an implicit or explicit agreement of reciprocity, practices have trust and justice underlying this contract (Rousseau, 1989; Dalal, 2005). There are two types of trust, cognition-based trust and affect-based trust, i.e., competence-based and integrity-based. In the first case, the one who violated the trust has an aspect of competence, and in the second, morality (Cropanzano et al., 2017). In this case, what was observed in the narratives was of the second type, moral. Thus, supervisor trust is related to more positive leadership (Schyns and Schilling, 2013), and trustworthy is related to reduced dishonesty, secrets, and knowledge hiding (Colquitt, 2001; Connelly et al., 2012) and to increased organizational citizenship and perceived ethical leadership (Xiao et al., 2020). Therefore, increased trust will promote an increase in positive responses with the manager (Cropanzano et al., 2017), however, it is unknown whether increased trust in the organization would also promote the same, given that there are no reports of perceptions of institutionalized practices or the HR management area by any of the organizational actors.

## Conclusion

This work provided support for different groups of HRMP already described in the literature, such as social exchange practices, communication practices, and occupational health practices (Van Beurden et al., 2020). These have reciprocity, consistency, and scarcity as their principle; however, the present study included one additional group of practices that come in the sense of bonding and friendship, one that is oriented to providing an environment in which health occurs because people like each other, because they trust each other, so it is related to a process of building social capital. These results also suggest that managers and workers have different perceptions of HRMP but do agree that social support is a frequent and relevant practice for protection and promotion of mental health at work.

## Data availability statement

The raw data supporting the conclusions of this article will be made available by the authors, without undue reservation.

## Ethics statement

The studies involving human participants were reviewed and approved by Research Ethics Committee of the Universidade de São Paulo number 4.342.411. The patients/participants provided their written informed consent to participate in this study.

## Author contributions

MS and AP-N: conceptualization. BS: data curation, funding acquisition, investigation, and resources. AP-N: formal analysis, validation, visualization, and writing–review and editing. BS, MS, and AP-N: methodology. MS: project administration and supervision. BS and MS: writing–original draft. All authors contributed to the article and approved the submitted version.

## Funding

This research was financially supported by the Coordenação de Aperfeiçoamento de Pessoal de Nível Superior (CAPES), Brazil (Finance Code 001). BS received a grant from the Coordination of Superior Level Staff Improvement (CAPES), number: 88887.600233/2021-00.

## Conflict of interest

The authors declare that the research was conducted in the absence of any commercial or financial relationships that could be construed as a potential conflict of interest.

## Publisher’s note

All claims expressed in this article are solely those of the authors and do not necessarily represent those of their affiliated organizations, or those of the publisher, the editors and the reviewers. Any product that may be evaluated in this article, or claim that may be made by its manufacturer, is not guaranteed or endorsed by the publisher.
